# Adipose–Muscle Crosstalk in COPD Cachexia: Early Adipose Atrophy Drives Subsequent Muscle Wasting

**DOI:** 10.1002/jcsm.70154

**Published:** 2025-12-09

**Authors:** Takashi Shimada, Shotaro Chubachi, Keisuke Nishikawa, Tetsuya Arai, Hideto Iizuka, Shiro Otake, Kaori Sakurai, Junko Hamamoto, Mamoru Sasaki, Tomoki Maetani, Naoya Tanabe, Katsunori Masaki, Hiroki Kabata, Jun Miyata, Yoshitake Yamada, Masahiro Jinzaki, Hidetoshi Nakamura, Koichiro Asano, Koichi Fukunaga

**Affiliations:** ^1^ Division of Pulmonary Medicine, Department of Medicine Keio University School of Medicine Tokyo Japan; ^2^ Department of Respiratory Medicine Japan Community Health Care Organization (JCHO) Saitama Medical Center Saitama Japan; ^3^ Department of Respiratory Medicine, Graduate School of Medicine Kyoto University Kyoto Japan; ^4^ Department of Radiology Keio University School of Medicine Tokyo Japan; ^5^ Department of Respiratory Medicine Saitama Medical University Saitama Japan; ^6^ Division of Pulmonary Medicine, Department of Medicine Tokai University School of Medicine Kanagawa Japan

**Keywords:** adipose tissue atrophy, cachexia, chronic obstructive pulmonary disease, muscle wasting

## Abstract

**Background:**

Chronic obstructive pulmonary disease (COPD) is frequently associated with cachexia, leading to poor prognoses and reduced quality of life. However, the mechanisms underlying adipose tissue atrophy, its pathological significance and its interaction with skeletal muscle remain poorly understood. We hypothesised that adipose tissue atrophy precedes muscle wasting in COPD‐associated cachexia, and muscle atrophy progresses through adipose–muscle crosstalk.

**Methods:**

We analysed chest computed tomography scans of 185 patients with COPD to quantify the cross‐sectional areas of the pectoralis muscle (PM), subcutaneous adipose tissue (SAT) and epicardial adipose tissue (EAT), and the percentage of low attenuation area (LAA%) as an index of emphysema. To elucidate the pathophysiological mechanisms underlying cachexia in COPD, we performed histological and molecular analyses of the lung, muscle and adipose tissues over time in a cigarette smoke–induced emphysema mouse model. Further, we used an in vitro culture system of differentiated adipocytes (3T3‐L1) and myotubes (C2C12) to study the effects of cigarette smoke extract (CSE) on adipose–muscle interaction.

**Results:**

In patients with COPD, the areas of PM, SAT and EAT all demonstrated significant negative correlations with LAA%; notably, PM and EAT were independently associated with the extent of emphysematous changes. In the smoke‐exposed murine model, adipose tissue atrophy was observed after 1 month of exposure, accompanied by increased expressions of IL‐6 and IL‐1β, macrophage infiltration and the upregulation of the lipolytic enzymes ATGL and HSL. The adipose atrophy had further progressed after 3 months of exposure, and the high expression of UCP1 was sustained, which suggested the browning of adipose tissue. Conversely, muscle atrophy was not evident at 1 month but became apparent after 3 months, coinciding with emphysema development. This was associated with the downregulation of the myogenic markers MyoD and Myogenin and the upregulation of the muscle degradation marker Atrogin‐1. In vitro experiments revealed that CSE exposure reduced lipid droplet content and induced IL‐6 and IL‐1β expressions in adipocytes. Conditioned media from CSE‐treated adipocytes triggered myotube atrophy and downregulated MyoD and Myogenin but upregulated Atrogin‐1.

**Conclusions:**

Our findings indicate that cigarette smoke‐induced adipose tissue atrophy precedes muscle wasting, and alterations in adipose tissue may contribute to muscle atrophy progression. Adipose tissue dysfunction may be implicated in the development of cachexia in patients with COPD, highlighting its potential as a therapeutic target.

## 1 Introduction

Chronic obstructive pulmonary disease (COPD) is characterised by airflow limitation resulting from airway inflammation and is pathologically defined by irreversible alveolar destruction, namely emphysema [1]. Cachexia, a condition marked by progressive loss of adipose tissue and skeletal muscle mass, is frequently observed in chronic illnesses such as malignancies and chronic kidney disease. COPD is also recognised as a critical underlying condition, probably due to the systemic spread of pulmonary inflammation induced by tobacco smoke exposure [2]. The development of cachexia in COPD is associated with poor prognosis [3] [S1], reduced quality of life [S2] and accelerated progression of emphysema [S3]. Thus, cachexia in COPD represents a severe clinical complication requiring preventive strategies and therapeutic interventions.

Previous studies, including ours, have demonstrated associations between body composition indices (such as fat‐free mass index [FFMI] and fat mass index [FMI]) and clinical outcomes in COPD [3, 4]. However, these indices do not allow detailed localisation of muscle and fat distribution. Chest computed tomography (CT) has been widely utilised for the routine clinical management of COPD, as well as the quantitative assessment of pulmonary and extrapulmonary structures [5]. The cross‐sectional area of the pectoralis muscle (PM) has been shown to correlate more strongly with COPD severity and prognosis than body mass index (BMI) [6]. Moreover, it has been reported that patients with COPD have higher epicardial adipose tissue (EAT) than healthy individuals [7]. However, no previous studies have objectively assessed the relationships between emphysema, airway indices and muscle or fat parameters and their potential disparities using CT‐based parameters.

The pathophysiology of cachexia in COPD has been mainly studied in the context of skeletal muscle. Studies involving human samples and animal models of tobacco smoke exposure have demonstrated muscle atrophy [8], enhanced muscle degradation [9] [S4] and impaired muscle synthesis [S5], with underlying mechanisms involving inflammation [S6], oxidative stress [S7] and upregulation of proteolytic systems such as the ubiquitin‐proteasome and lysosomal pathways [9, 10]. In contrast, the role of adipose tissue loss and its involvement in the pathogenesis of cachexia in COPD remains underexplored [S8]. A single study using a tobacco smoke‐exposed mouse model showed that smoke exposure resulted in adipose tissue atrophy, adipocyte shrinkage, systemic inflammation and macrophage infiltration within adipose tissue [11]. In cancer cachexia, adipose tissue loss is well established as an early and crucial event [12], with emerging evidence suggesting that adipose depletion contributes to muscle wasting through interaction between the two tissues [13]. However, the mutual mechanisms linking adipose tissue loss and muscle atrophy in COPD‐associated cachexia remain to be elucidated. Based on these findings, we hypothesised that smoke‐induced early adipose tissue depletion initiates subsequent muscle wasting through reciprocal pathological crosstalk.

Elucidating the pathogenesis of cachexia and developing therapeutic interventions becomes extremely difficult once it progresses and substantial physical dysfunction occurs. Therefore, it is essential to investigate the early structural abnormalities and molecular mechanisms that precede overt cachexia [14]. Smoke‐induced emphysema mouse models enable longitudinal analysis of muscle and adipose tissues before and after emphysema development, an approach that is not feasible in human studies. Furthermore, in vitro cell culture experiments are essential to directly study the interactions between adipocytes and myotubes, given the presence of multiple cell types beyond myofibres and adipocytes within these tissues [15] [S9] and the complexity of interorgan signalling in vivo.

The objectives of the present study were (a) to analyse chest CT images of patients with COPD to assess the associations between pulmonary emphysema, airway pathology and morphologic changes in muscle and adipose tissues; (b) to use a smoking‐induced emphysema mouse model to assess the longitudinal pathologic and molecular changes in adipose and muscle tissues under smoking exposure; and (c) to use adipocyte and myotube cell lines to directly evaluate the intercellular interactions between adipose and muscle tissues under smoking conditions to elucidate the role of adipose tissue loss and its crosstalk with muscle wasting in COPD‐associated cachexia.

## 2 Methods

### 2.1 Imaging Analysis of Patients With COPD

**FIGURE 1:**
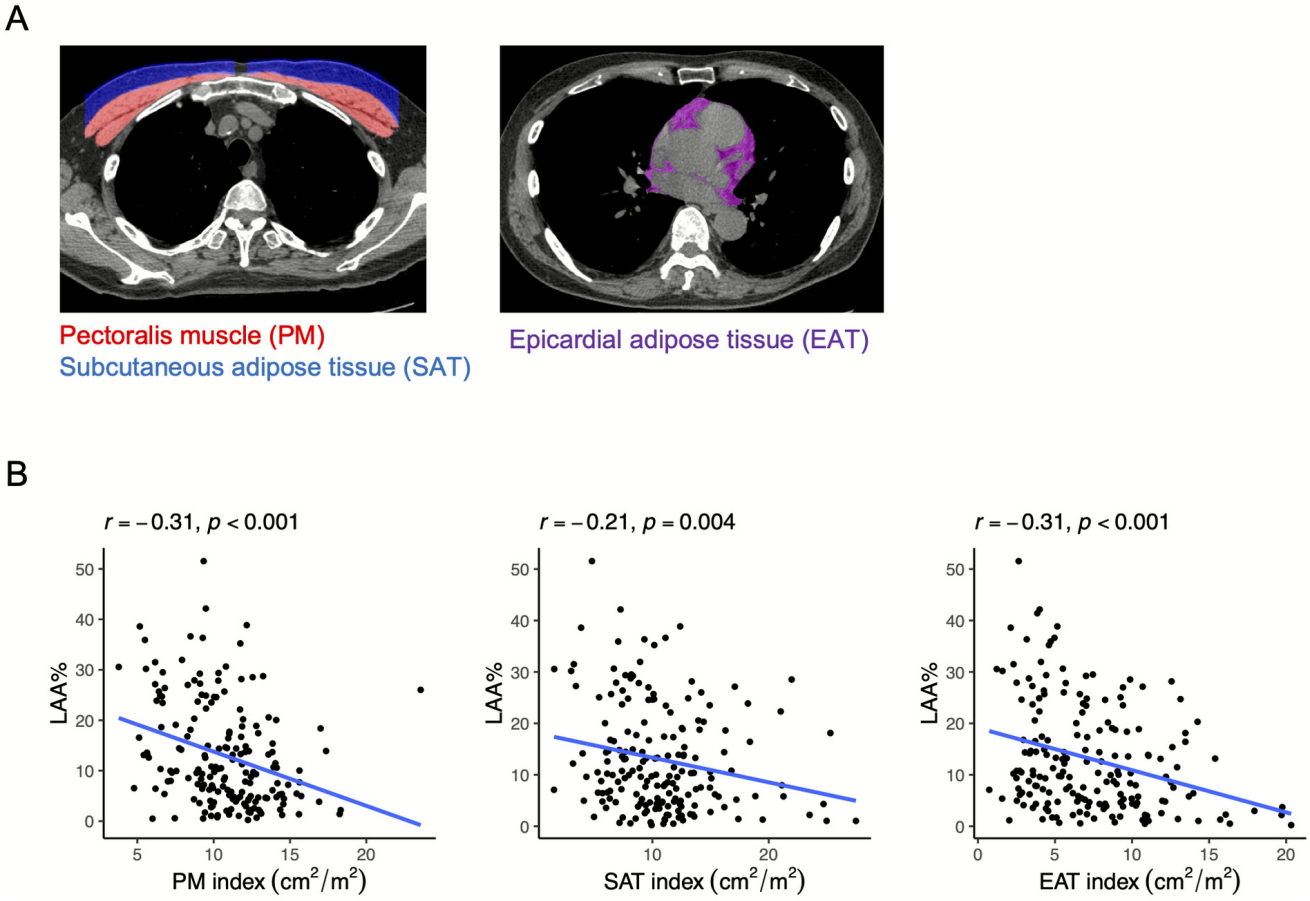
Representative CT images used for measurement of muscle and adipose tissues, and correlations between cross‐sectional areas of muscle/adipose tissues and emphysema severity in patients with COPD. (A) Measurement of the pectoralis muscle (PM, red), subcutaneous adipose tissue (SAT, blue), and epicardial adipose tissue (EAT, pink) on a CT image. (B) Correlation between cross‐sectional areas of muscle/adipose tissues and quantitative emphysema severity in patients with COPD. COPD, chronic obstructive pulmonary disease; LAA%, ratio of low attenuation area.

As described in the Supporting Information Methods, this study involved 185 patients diagnosed with COPD by spirometry who had available non‐contrast chest CT data (Figure S1). For the quantification of muscle and adipose tissue, the ImageJ software was used for manual masking of regions of interest, and a custom Python script was used for automatic area calculation. The PM was manually segmented on an axial slice just above the aortic arch by selecting regions with CT attenuation values between −50 and +90 Hounsfield units (HU) [5]. Subcutaneous adipose tissue (SAT) of the chest was automatically identified within the area between the PM and the skin surface on the same axial slice [5]. The EAT was delineated by manually tracing the pericardium at the level of the origin of the left coronary artery and extracting regions within a CT attenuation range of −230 to −30 HU [16] (Figure 1A). The PM index, SAT index and EAT index were calculated by dividing the cross‐sectional area (centimetres squared) of PM, SAT and EAT by the square of the height (meters squared) to account for inter‐individual differences in body size [5]. The percentage of low attenuation area (LAA%) and airway wall area (WA%) were quantified using custom‐made software (AZE Ltd., Tokyo, Japan) [4], while the total airway count (TAC) and airway fractal dimension (AFD) were calculated using a custom Python script [17, 18].

### 2.2 Mouse Model

Female C57BL/6J mice (7–8 weeks old) were exposed to cigarette smoke and intratracheal elastase administration as described in the Supporting Information Methods. Mice were euthanised at either 4 or 12 weeks after the initiation of cigarette smoke exposure or 3 weeks after elastase instillation. Lung, adipose tissue and skeletal muscle samples were collected for morphological evaluation. Tissues were fixed in 4% paraformaldehyde (PFA), followed by processing, paraffin embedding, sectioning and histopathologic analysis as described in the Supporting Information Methods.

### 2.3 Reverse Transcription Polymerase Chain Reaction (RT‐PCR)

RNA extraction and reverse transcription were performed following the methods specified in the Supporting Information Methods. RT‐PCR was performed using the QuantStudio 5 Real‐Time PCR System (Thermo Fisher Scientific, Waltham, MA, United States) with SYBR Green chemistry. GAPDH was used as the internal control for normalisation. Primer sequences used for PCR amplification are provided in Table S1.

### 2.4 Protein Extraction and Western Blotting

Mouse gastrocnemius muscle and C2C12 (CRL‐1772; ATCC, Manassas, VA, United States) myotubes were lysed in a radioimmunoprecipitation assay (RIPA) buffer (Thermo Fisher Scientific) according to the protocol of the manufacturer. Equal amounts of protein (10 μg per lane) were subjected to SDS‐PAGE and Western blotting, as described in the Supporting Information Methods.

### 2.5 Cell Line Experiments

Murine preadipocytes 3T3‐L1 (CL‐173; ATCC) and skeletal muscle myoblasts C2C12 were cultured according to the protocols of the manufacturer, as detailed in the Supporting Information Methods. Differentiated 3T3‐L1 adipocytes were treated with 3% cigarette smoke extract (CSE) prepared as described in the Supporting Information Methods, and the supernatant was collected after 24 h, for the preparation of adipocyte‐conditioned medium supplemented with CSE (Adipose + CSE). Control supernatants were obtained by incubating 3% CSE for 24 h in the absence of cells. All supernatants were filtered through 0.22 μm membranes and stored at −80°C. Adipose + CSE or CSE alone was diluted 1:3 with myotube differentiation medium and applied to C2C12 myotubes with medium changes every 48 h. Cells were harvested after 96 h of treatment. All experiments were independently repeated at least three times, and the representative results are presented.

### 2.6 Immunofluorescent Staining for Cells

To assess lipid droplet accumulation, differentiated 3T3‐L1 adipocytes were fixed with 4% PFA and stained with Nile Red (72 485; Sigma‐Aldrich, St. Louis, MO, United States), and the stained area was quantified using the ImageJ software. For the evaluation of myotubes, C2C12 cells were fixed with 4% PFA and stained with an anti‐myosin heavy chain (MyHC) antibody (MAB4470; R&D Systems, Minneapolis, MN, United States). The myotube diameter was measured using the ImageJ software. Nuclei were counterstained with DAPI, and slides were mounted with Vectashield mounting medium (Vector Laboratories, Newark, CA, United States).

### 2.7 Statistical Analysis

Data are presented as mean ± standard error (SE) unless otherwise indicated. Inter‐group comparisons were performed using Student's *t*‐test. To assess the relationship between extrapulmonary organ measurements and emphysema on chest CT, multivariate linear regression analyses were performed with adjustment for age, sex, current smoking status and smoking index. Correlations between continuous variables were evaluated using Pearson's correlation coefficient. All statistical analyses were performed with JMP Pro version 17 (SAS Institute, Cary, NC, United States). Two‐sided *p*‐values were reported, and statistical significance was defined as *p* < 0.05.

## 3 Results

### 3.1 Imaging Analysis of Patients With COPD

The baseline characteristics of the 185 patients with COPD enrolled in this study are summarised in Table 1. Their mean age was 72.5 ± 8.0 years, and 9.7% were females. COPD was classified as GOLD stages 1, 2, 3 and 4 for 33.5%, 44.9%, 17.3% and 4.3% of the patients, respectively [1]. Figure 1B shows the correlations between PM, SAT, EAT and LAA%. All three indices (PM, SAT and EAT) demonstrated significant correlations with LAA%, whereas airway indices such as WA%, TAC and AFD did not show significant associations (Table S2). Multivariate analysis adjusted for age, sex, current smoking status and smoking index is presented in Table 2. PM, SAT and EAT were independently associated with LAA%, and both PM and EAT showed independent correlations with LAA%. These results suggest that reductions in the pectoral muscle and thoracic adipose areas, especially visceral adiposity as reflected by EAT, are associated with greater emphysematous changes.

**TABLE 1:** Patients characteristics.

Parameter	Total
	(*n* = 185)
Age, years	72.5 ± 8.0
Sex, female (%)	18 (9.7)
Current smoking (%)	17 (9.2)
Packyear	52.9 ± 31.1
BMI, kg/m^2^	23.1 ± 3.2
FMI, kg/m^2^	5.3 ± 2.0
FFMI, kg/m^2^	17.9 ± 2.0
GOLD grade 1/2/3/4, (%)	62/83/32/8
	(33.5/44.9/17.3/4.3)
%VC, %	98.0 ± 17.4
%FEV_1_, %	68.0 ± 20.6
LAA%, %	61.3 ± 23.1
WA%, %	39.6 ± 8.4
TAC	154.9 ± 90.0
AFD	1.67 ± 0.10
PM index, cm^2^/m^2^	10.7 ± 3.0
SAT index, cm^2^/m^2^	10.7 ± 4.6
EAT index, cm^2^/m^2^	7.2 ± 3.9

*Note:* Data are shown as n (%) or mean ± SD.

Abbreviations: %FEV_1_, ratio of predicted forced expiratory volume in 1 s; %VC, ratio of predicted vital capacity; AFD, airway fractal dimension; BMI, body mass index; EAT, epicardial adipose tissue; FFMI, fat‐free mass index; FMI, fat mass index; GOLD, global initiative for chronic obstructive lung disease; LAA%, ratio of low attenuation area; PM, pectoralis muscles; SAT, subcutaneous adipose tissue; SD, standard deviation; TAC, total airway count.

**TABLE 2:** Multivariable analysis of the cross‐sectional area of muscle and adipose tissue and the quantification of emphysema in patients with COPD.

	Model 1		Model 2		Model 3		Model 4	
	β	*p*‐value	β	*p*‐value	β	*p*‐value	β	*p*‐value
PM index	−0.36	< 0.001	—	—	—	—	−0.29	< 0.001
SAT index	—	—	−0.27	< 0.001	—	—	0.067	0.44
EAT index	—	—	—	—	−0.45	< 0.001	−0.42	< 0.001
Age	0.018	0.8	0.035	0.63	0.18	0.015	0.15	0.037
Sex	−0.15	0.047	0.037	0.62	0.006	0.93	−0.13	0.08
Current smoking	0.033	0.64	0.064	0.38	0.053	0.44	0.02	0.77
Packyear	0.079	0.26	0.12	0.11	0.16	0.021	0.16	0.019

Abbreviations: COPD, chronic obstructive pulmonary disease; EAT, epicardial adipose tissue; PM, pectoralis muscles; SAT, subcutaneous adipose tissue.

### 3.2 Time Course of Alveolar Destruction, Muscle and Adipose Tissue in Smoking and Elastase‐Induced Emphysema Models

**FIGURE 2:**
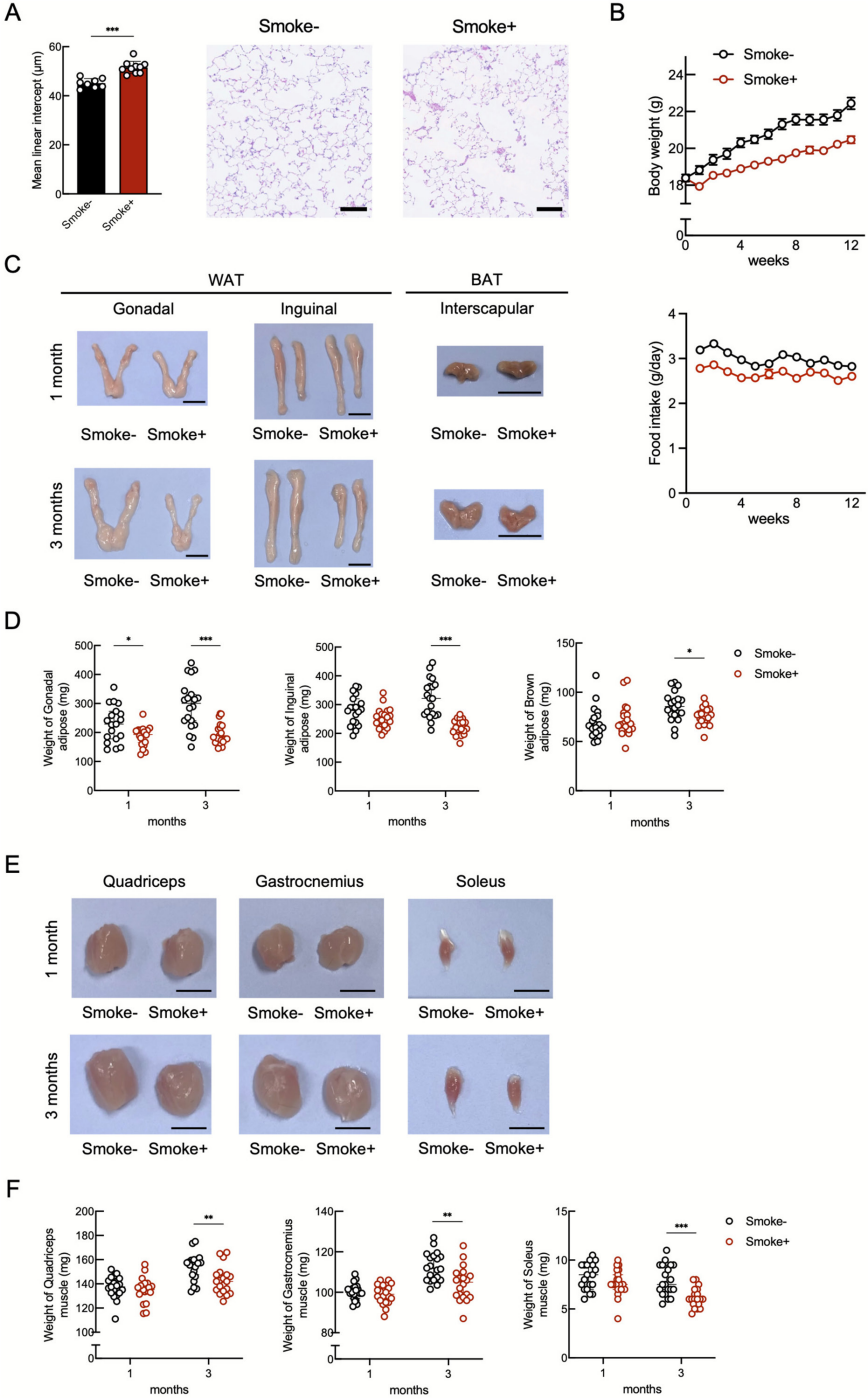
Pulmonary, adipose and skeletal muscle alterations in a cigarette smoke exposure mouse model. (A) Representative H&E‐stained lung sections and evaluation of emphysematous changes (*n* = 8–10 per group). Scale bar: 100 μm. (B) Changes in body weight and food intake over 3 months. (C) Representative macroscopic appearance of adipose tissue. Scale bar: 1 cm. (D) Comparison of adipose tissue weight (*n* = 19–20 per group). (E) Representative macroscopic appearance of skeletal muscle. Scale bar: 5 mm. (F) Comparison of skeletal muscle weight (*n* = 19–20 per group). *p < 0.05; **p < 0.01; ***p < 0.001; BAT, brown adipose tissue; WAT, white adipose tissue.

The changes in alveolar indices, body weight, food intake, adipose tissue and muscle mass in the smoking‐induced emphysema model are shown in Figure 2. The mean alveolar diameter was significantly larger for the smoking group relative to the nonsmoking group after 3 months of smoke exposure (Figure 2A). Body weight consistently remained lower for the smoking group throughout the smoking period. Also, mice in the smoking group demonstrated a modest reduction in food intake compared with those in the nonsmoking group (Figure 2B). Visceral fat mass was visibly and quantitatively reduced for the smoking group relative to the controls after 1 month of exposure. The reductions in visceral, subcutaneous and brown adipose tissues (BATs) were evident macroscopically and by measuring tissue weight at 3 months (Figure 2C,D). In contrast, the quadriceps, gastrocnemius and soleus muscles showed no significant differences at 1 month, but exhibited significant atrophy after 3 months of exposure (Figure 2E,F). The alveolar diameter was significantly increased in the elastase‐induced emphysema model, but the body weight, fat mass and muscle mass did not differ significantly from those of the controls (Figure S2). These results indicate that smoking has systemic effects leading to muscle and fat atrophy that are independent of direct alveolar destruction, and fat loss precedes muscle atrophy during the early stages of smoking exposure.

### 3.3 Adipose Tissue Alterations in Smoking‐Exposed Mice

**FIGURE 3:**
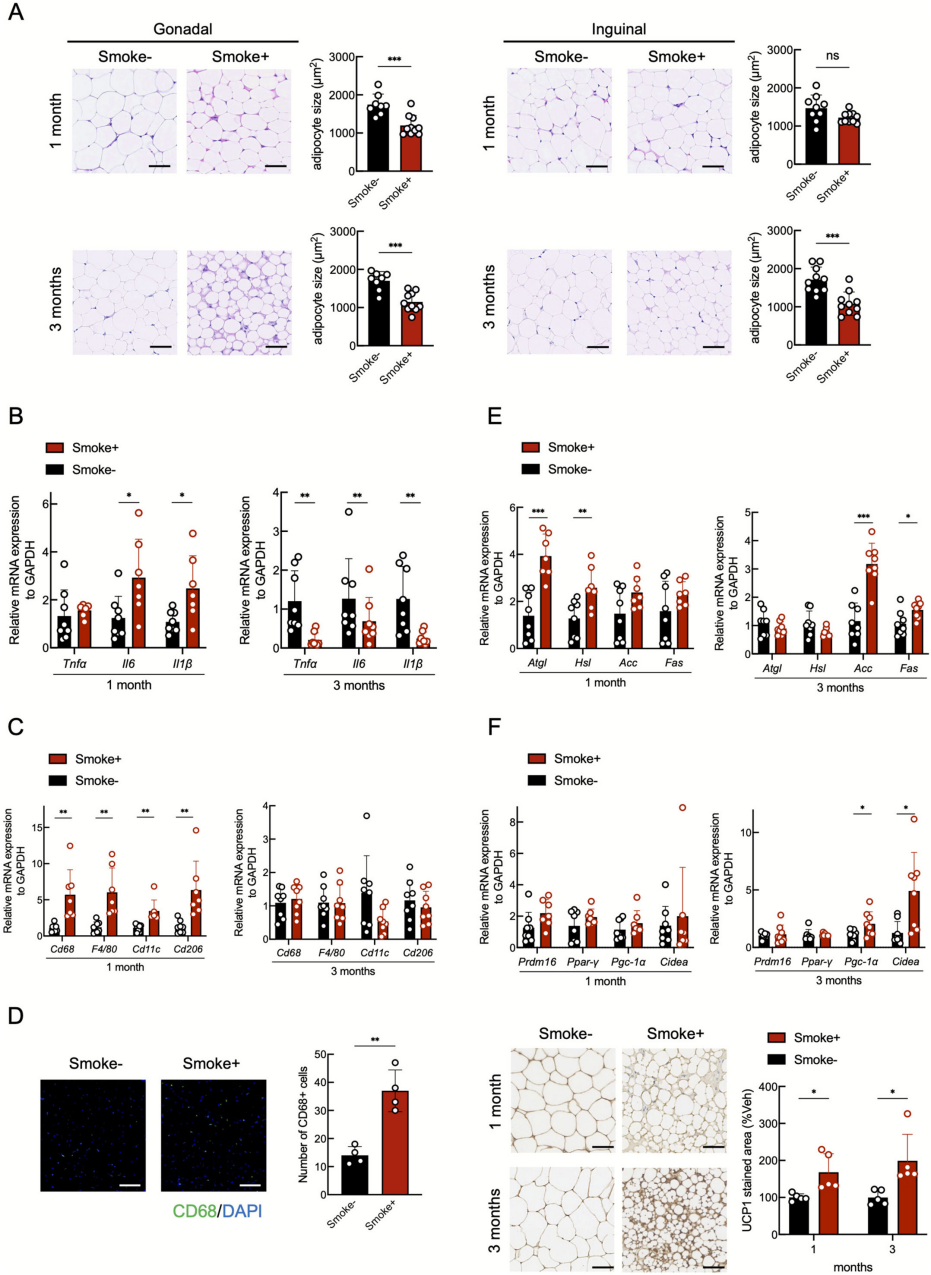
Cigarette smoke‐induced adipose tissue atrophy in mice. (A) Histological comparison of perigonadal and subcutaneous adipose tissue in mice (*n* = 8–10 per group). Scale bar: 50 μm. (B) RT‐PCR analysis of proinflammatory cytokines in mouse visceral adipose (*n* = 7–8 per group). (C) RT‐PCR analysis of macrophage markers in visceral adipose (*n* = 7–8 per group). (D) Immunofluorescence analysis of CD68 expression in visceral adipose tissue under moderate‐power fields after 1 month of smoke exposure (*n* = 4 per group). Scale bar: 100 μm. (E) RT‐PCR of lipolytic and lipogenic genes in visceral adipose (*n* = 7–8 per group). (F) RT‐PCR of browning markers in visceral adipose (*n* = 7–8 per group) and immunohistochemical detection of UCP1 expression in visceral adipose (*n* = 5 per group). Scale bar: 50 μm. *p < 0.05; **p < 0.01; ***p < 0.001; ns, not significant.

The temporal changes in adipose tissue during smoking exposure are shown in Figure 3. In the smoking group, visceral adipocyte size was significantly reduced at 1 and 3 months (Figure 3A). The gene expressions of the inflammatory markers IL‐6 and IL‐1β were elevated at 1 month but declined by 3 months (Figure 3B). Furthermore, the expressions of the macrophage markers CD68, F4/80, CD11c and CD206 were upregulated at 1 month, with immunofluorescence showing a significantly greater number of CD68‐positive cells in the smoking group (Figure 3C,D). The lipolytic enzymes ATGL and HSL were upregulated at 1 month, whereas the expressions of the lipogenic enzymes ACC and FAS were increased at 3 months (Figure 3E). Smoking exposure also induced browning of adipose tissue, as evidenced by the upregulation of browning‐related genes and increased UCP1 positive staining observed at 1 and 3 months (Figure 3F). Collectively, these results indicate that early smoking exposure induces adipocyte atrophy, inflammation, lipolysis and browning, followed by compensatory lipogenesis and inflammation resolution, although adipose atrophy and browning appear to be irreversible.

### 3.4 Skeletal Muscle Alterations in Smoking‐Exposed Mice

**FIGURE 4:**
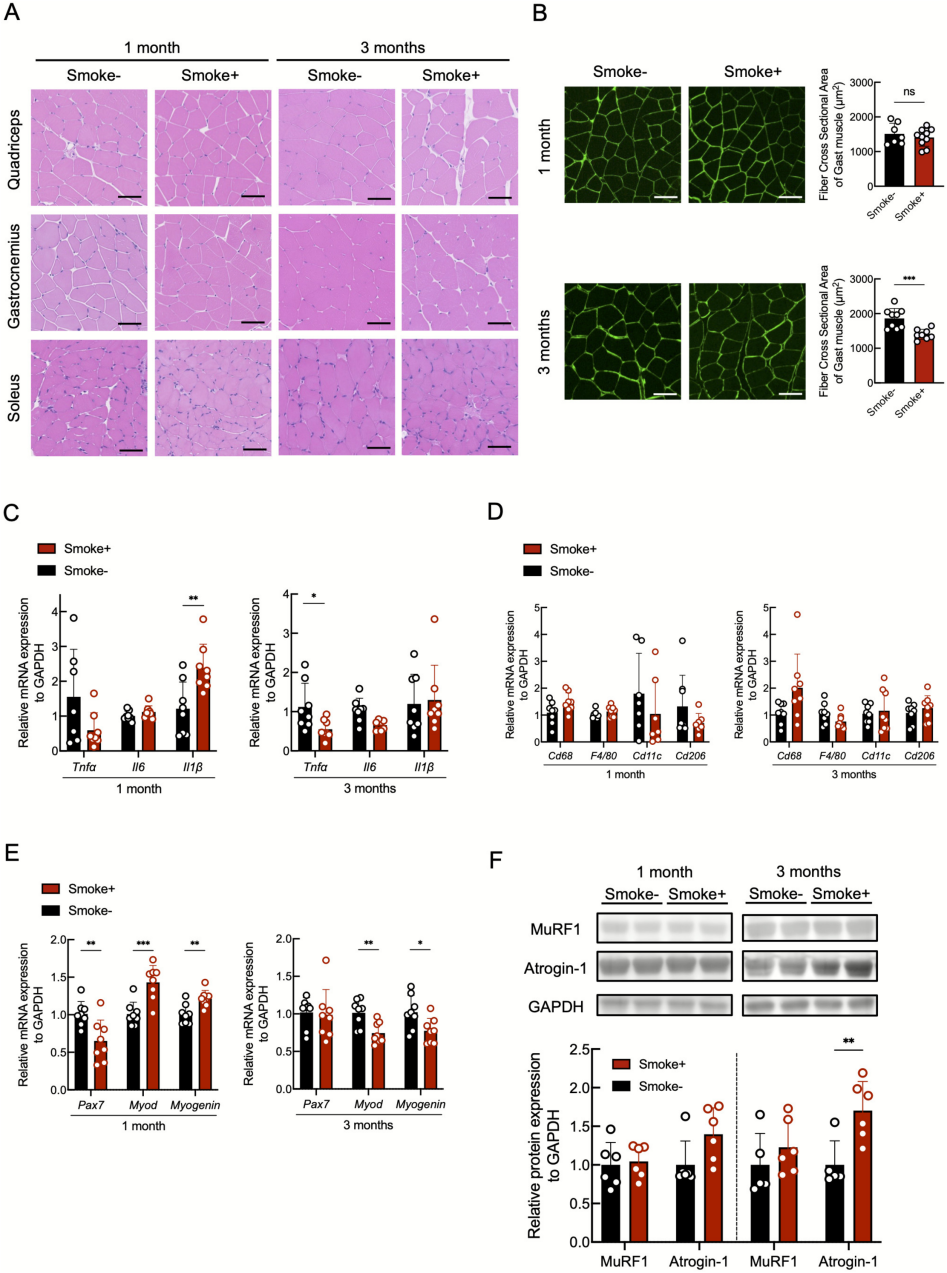
Cigarette smoke‐induced skeletal muscle atrophy in mice. (A) Histological comparison of skeletal muscle tissue (*n* = 7–10 per group). Scale bar: 50 μm. (B) Immunofluorescence analysis of laminin expression in gastrocnemius muscle (*n* = 5 per group). Scale bar: 50 μm. (C) RT‐PCR of inflammatory cytokines in gastrocnemius muscle (*n* = 7–8 per group). (D) RT‐PCR of macrophage markers in gastrocnemius muscle (*n* = 7–8 per group). (E) RT‐PCR of myogenic regulatory factors in gastrocnemius muscle (*n* = 8 per group). (F) Immunoblot analysis of muscle atrophy markers in gastrocnemius lysates (*n* = 5–6 per group). *p < 0.05; **p < 0.01; ***p < 0.001; ns, not significant.

Temporal changes in skeletal muscle during smoking exposure are shown in Figure 4. While no gross changes were observed at 1 month, the smoking group had visible muscle atrophy and decreased muscle fibre cross‐sectional area as assessed by laminin staining at 3 months (Figure 4A,B). Inflammatory evaluation revealed that IL‐1β expression was elevated at 1 month, whereas TNF‐α expression decreased at 3 months (Figure 4C). Analysis of muscle anabolic and catabolic factors showed that the expressions of the anabolic factors MyoD and Myogenin were upregulated at 1 month, but both were significantly downregulated at 3 months for the smoking group (Figure 4E). The catabolic factors MuRF1 and Atrogin‐1 were unchanged at 1 month, but Atrogin‐1 expression was significantly increased at 3 months in the smoking group (Figure 4F). These results suggest that late stages of smoking exposure are characterised by decreased anabolic signalling, increased catabolic signalling and overt muscle wasting although the early stages do not lead to overt muscle atrophy despite concurrent fat loss.

### 3.5 Adipocyte–Myotube Crosstalk Under Smoking Conditions: In Vitro Analysis

**FIGURE 5:**
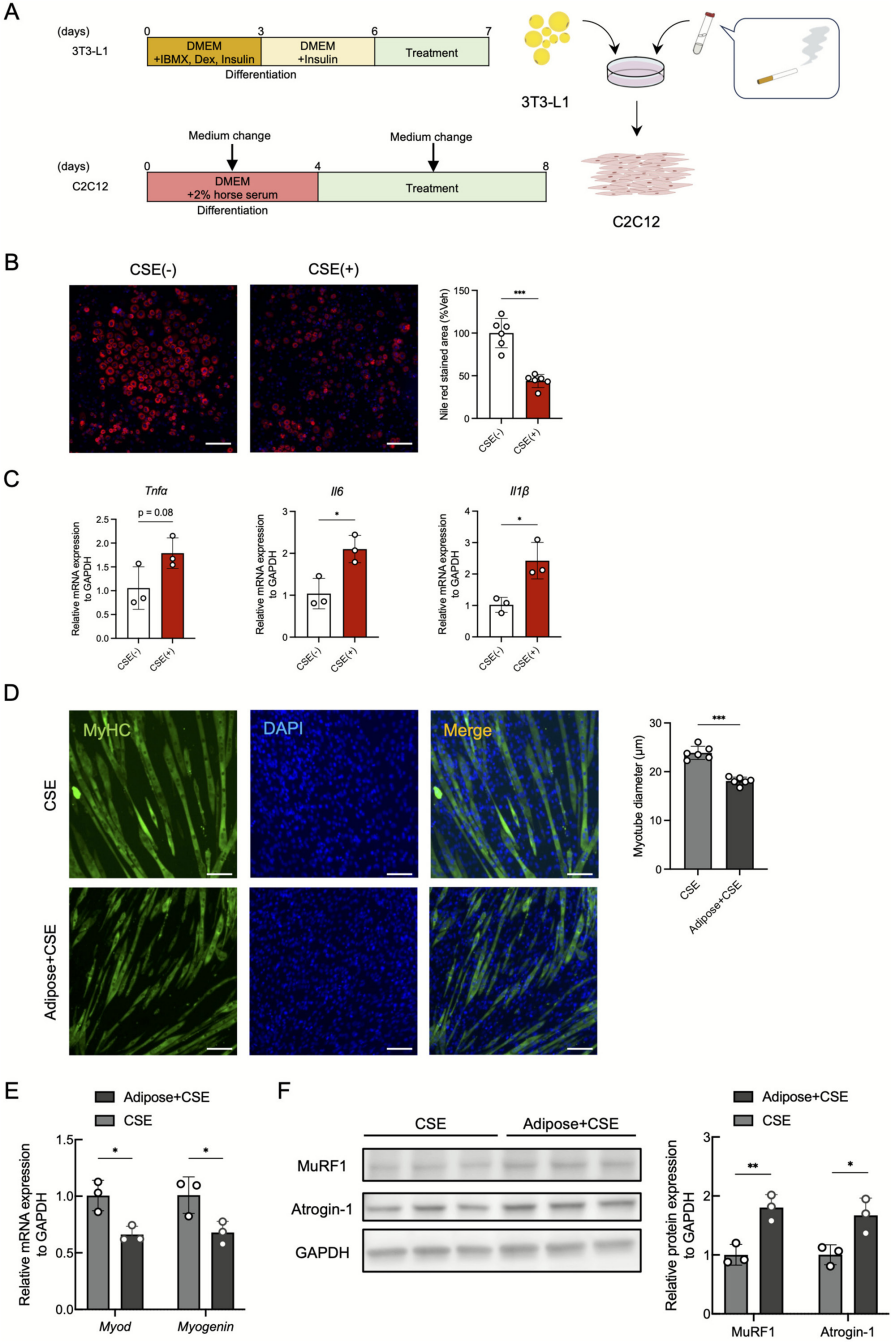
In vitro cellular experiments. (A) Schematic of experimental design. (B) Nile red staining of differentiated 3T3‐L1 adipocytes (*n* = 6 per group). Scale bar: 200 μm. (C) RT‐PCR analysis of proinflammatory cytokines in differentiated 3T3‐L1 adipocytes (*n* = 3 per group). (D) Immunofluorescence staining of MyHC (green) and DAPI (blue) in C2C12 myotubes (*n* = 6 per group). Scale bar: 100 μm. (E) RT‐PCR analysis of myogenic regulatory genes in C2C12 myotubes (*n* = 3 per group). (F) Immunoblot analysis of atrophy markers in C2C12 myotubes (*n* = 3 per group). *p < 0.05; **p < 0.01; ***p < 0.001; CSE, cigarette smoke extract; MyHC, myosin heavy chain; ns, not significant.

Based on the observation that adipose tissue atrophy precedes muscle atrophy after smoking exposure, the adipocyte–myotube interaction under smoking conditions was examined in cellular models. The 3T3‐L1 adipocytes were treated with CSE, and their conditioned medium was subsequently applied to C2C12 myotubes (Figure 5A). CSE exposure resulted in a decrease in Nile red positive lipid droplets and an increase in inflammatory cytokines in 3T3‐L1 adipocytes (Figure 5B,C). Treatment of C2C12 myotubes with Adipose + CSE medium resulted in a greater reduction in the MyHC‐positive myotube diameter than with CSE alone (Figure 5D). Moreover, MyoD and Myogenin expression levels were lower in myotubes exposed to Adipose + CSE medium, whereas the protein expressions of atrophy markers MuRF1 and Atrogin‐1 were upregulated (Figure 5E,F). These results suggest that cigarette smoke directly induces adipocyte atrophy, and smoke‐induced adipocyte dysfunction exacerbates muscle atrophy by suppressing myogenic factors and enhancing muscle catabolic pathways.

## 4 Discussion

**FIGURE 6:**
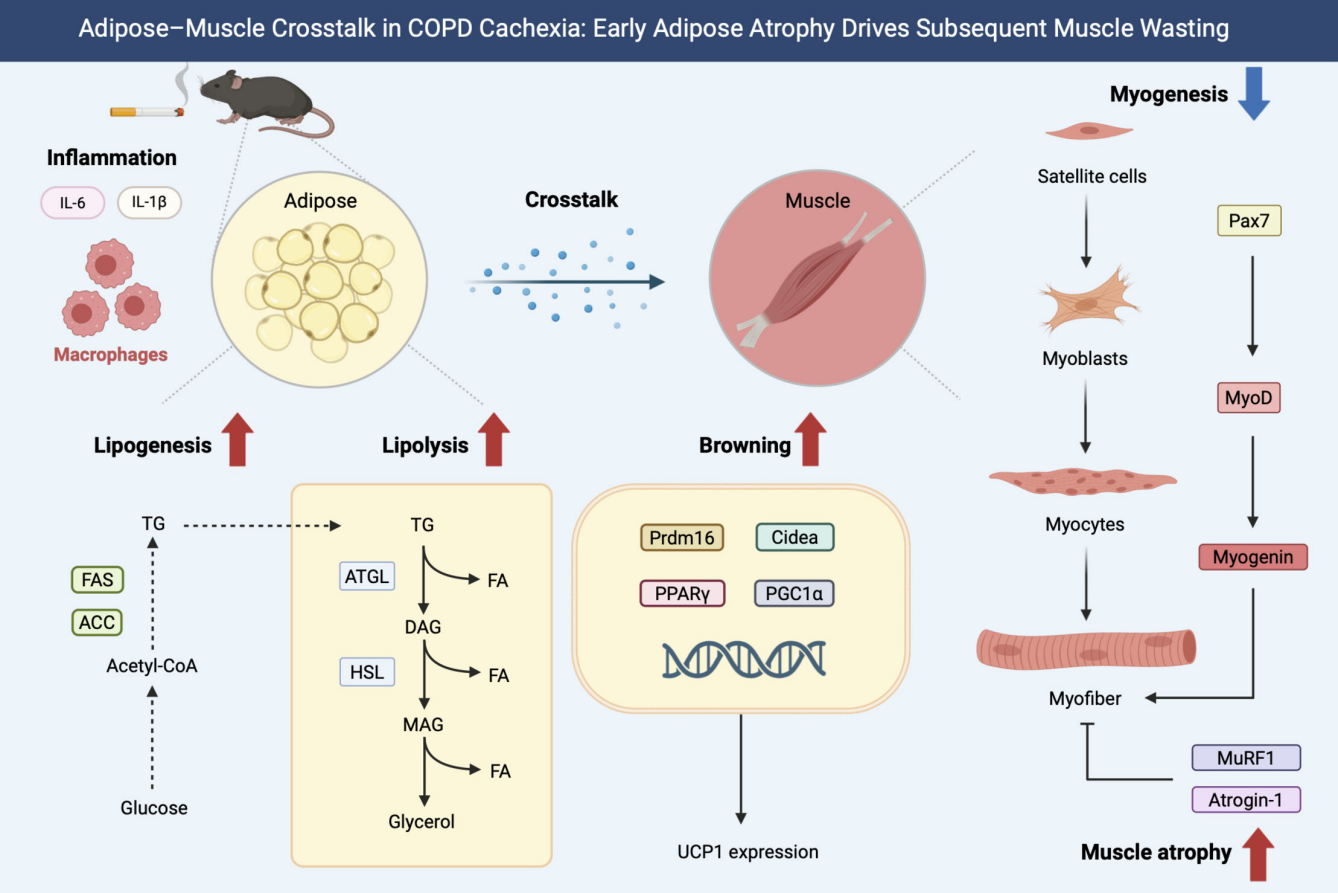
Schematic representation of adipose and muscle tissue dynamics in a cigarette smoke‐induced emphysema mouse model. In the cigarette smoke‐induced emphysema mouse model, early exposure to cigarette smoke resulted in the marked reduction in adipose tissue mass, followed by the onset of muscle atrophy at later stages. In adipose tissue, cigarette smoke exposure induced inflammatory changes, lipolysis and browning, accompanied by a compensatory upregulation of adipogenesis. In muscle tissue, the loss of adipose tissue was followed by a reduction in muscle mass, characterised by decreased muscle protein synthesis and overt muscle atrophy. Our findings suggest that adipose tissue atrophy precedes muscle wasting after cigarette smoke exposure, implicating that smoke‐induced alterations in adipose tissue properties as a potential contributor to the onset of muscle degeneration. DAG, diacylglycerol; FA, fatty acids; MAG, monoacylglycerol; TG, triacylglycerol.

This study elucidated the dynamics and interplay between adipose and muscle tissues in the context of COPD using an integrated approach combining chest CT image analysis of human patients with COPD, a murine COPD model and in vitro cellular assays. We demonstrated that adipose and skeletal muscle tissues are associated with emphysema severity in patients with COPD. Our results indicate that adipose atrophy precedes muscle wasting in response to cigarette smoke exposure, and smoking‐induced changes in adipose tissue properties may contribute to the onset of muscle atrophy (Figure 6). A major strength of this study is its multifaceted design; it integrates clinical imaging, animal models and cellular biology. The results emphasise the clinical significance of assessing adipose and muscle dynamics in patients with COPD and suggest that identifying specific targets for intervention may contribute to the prevention and, ultimately, the treatment of cachexia with meaningful clinical implications.

CT image analysis for patients with COPD revealed a negative correlation between LAA% and all of the following: PM, SAT and EAT. Multivariate analysis further indicated that PM and EAT were independently associated with LAA%. Previous studies have identified reduced PM mass as a poor prognostic factor [6] and are associated with impaired lung function and emphysema progression [19]. EAT has also been shown to negatively correlate with FEV_1_ and emphysema severity [20], supporting our findings that adipose and muscle tissues are involved in emphysema progression. PM and EAT were independently associated with disease progression, whereas SAT showed no correlation. Visceral fat is more proximal to the pulmonary artery than subcutaneous fat, making it more susceptible to inflammation propagated through the pulmonary circulatory system in response to cigarette smoke [21]. Given the widespread clinical use of chest CT in COPD management, the ability to simultaneously assess emphysema severity and body composition enhances its utility as a biomarker.

Elastase administration did not induce cachexia in mice, but chronic cigarette smoke exposure did, suggesting that cachexia results from the systemic effects of smoking rather than structural lung damage alone. In our smoke‐induced mouse model, adipose tissue atrophy preceded muscle wasting; early inflammatory responses and lipolysis in adipose tissue were followed by delayed muscle atrophy corresponding to the emergence of emphysematous changes. These findings are consistent with previous reports by studies of cancer cachexia involving humans [22] and mouse models [14], where adipose atrophy also preceded muscle wasting. Temporal investigations into muscle and adipose dynamics in cigarette smoke‐exposed mice remain scarce. Previous research has reported fat loss after 72 days of exposure [11], but longitudinal trajectories were not detailed. One study that evaluated muscle chronologically in smoke‐exposed mice found mild atrophy at 8 and 16 weeks, with more pronounced atrophy at 24 and 32 weeks. Interestingly, microarray analysis revealed the most significant pathway dysregulation in muscle tissue at 16 weeks [23]. These findings are consistent with our results and suggest that the pathophysiology of smoking‐induced cachexia is dynamic and requires longitudinal investigation. Moreover, in this study, food intake was lower in smoking‐exposed mice than that in controls. Consistent with this observation, previous studies have reported that appetite and food consumption increase following smoking cessation compared with during active smoking [S10]. These findings suggest that, in addition to its systemic effects, smoking‐induced reductions in appetite and food intake may also contribute to the development of cachexia.

After 1 month of cigarette smoke exposure, we observed the upregulation of IL‐6, IL‐1β and key lipolytic enzymes ATGL and HSL. By 3 months, this inflammatory and lipolytic response had subsided, while the expressions of lipogenic markers ACC and FAS increased. Significant macrophage infiltration was detected in adipose tissue after 1 month of exposure. Adipose tissue atrophy can result from increased lipolysis and decreased lipogenesis, with ATGL and HSL being key regulators of fat degradation [S11]. IL‐6 and IL‐1β have also been shown to directly promote lipolysis [S12] [S13] [S14], suggesting their involvement in this process. The principal sources of IL‐6 and IL‐1β in this study remain unclear, but adipocytes and macrophages are major cytokine producers within adipose tissue [15] [S15]. Thus, these cells are likely contributors. Alternatively, the systemic spillover of inflammatory mediators and immune cells associated with COPD [24] may be involved. Our in vitro data showed that CSE directly induced IL‐6 and IL‐1β expressions. Furthermore, previous studies have demonstrated that increased lipolysis acts as a chemotactic signal for macrophages [25], supporting the former mechanism. Inflammatory cytokines increased in adipose tissue during the early phase but decreased later. This is consistent with previous studies reporting no elevation of inflammatory markers in the adipose tissues of patients with COPD who have cachexia [26] and reduced cytokine concentrations in murine adipose tissue under hypoxic conditions [27], highlighting the dynamic nature of adipose tissue inflammation.

Adipose tissue is broadly classified into white adipose tissue (WAT) that is primarily involved in energy storage and BAT that is specialised for thermogenesis [S16]. Recent studies involving various models of disease‐associated cachexia have demonstrated that browning of WAT occurs at an early stage of cachexia, contributing to increased thermogenic activity and progression of the condition [28] [S17] [S18]. These browning adipocytes, also referred to as ‘beige cells’, are characterised by a high mitochondrial content and increased expression of UCP1 [29]. In this study, UCP1 expression was consistently elevated regardless of the duration of cigarette smoke exposure, suggesting an increase in beige cell presence within adipose tissue. Previous literature has reported that browning of adipose tissue precedes skeletal muscle atrophy in cancer‐associated cachexia [30]. Browning was observed before the onset of muscle atrophy. Inflammation and lipolysis had subsided during the later stages of emphysema, yet browning remained evident. Previous studies have shown that thermogenesis‐driven browning can lead to adipose tissue atrophy [30], suggesting that browning rather than inflammation may be a key contributor to adipose loss during the chronic phase. We further postulate the mechanisms underlying browning in this model. Given the early upregulation of lipolytic factors following smoke exposure, it is plausible that localised increases in free fatty acids induced UCP1 expression and subsequent browning [31]. The sustained browning observed during the later phases suggests additional regulatory mechanisms. Recent advances have permitted the elucidation of the mechanisms underlying cachexia‐induced browning [29], and emerging evidence suggests that factors involved in browning may serve as potential therapeutic targets for sarcopenia [14]. Future investigations are warranted to elucidate the molecular mechanisms driving adipose browning in COPD‐associated cachexia.

In our study, skeletal muscle atrophy was observed during the late phase of emphysema, which is consistent with previous findings in long‐term smoking mouse models [23, 32]. Gene expressions of the myogenic regulatory factors MyoD and Myogenin were upregulated during the early phase of cigarette smoke exposure, but downregulated during the later phase. These findings suggest that the capacity for muscle protein synthesis is preserved during the early period of smoke exposure, which is consistent with the absence of observable muscle atrophy at that time. The ubiquitin‐proteasome system is a principal pathway implicated in smoke‐induced muscle wasting and degeneration [9, 10], with MuRF1 and Atrogin‐1 being key regulatory factors mediating protein catabolism via this mechanism [S19]. Consistent with these findings, increased expression of Atrogin‐1 was observed in muscle tissue during the late phase of our model. Pro‐inflammatory cytokines such as TNF‐α and IL‐6 are known to enhance protein degradation while suppressing synthesis in muscle tissue [S20]. Inflammatory cytokine upregulation in muscle tissue was relatively mild relative to that in adipose tissue in our study. This suggests that mechanisms beyond direct inflammation, possibly involving inter‐tissue communication, may underlie muscle wasting.

Our in vitro experiments revealed that cigarette smoke‐induced atrophied adipocytes contributed to muscle atrophy. Adipose–muscle crosstalk has been implicated in cancer cachexia, where dysregulated adipocyte function initiates muscle loss [33]. Inhibiting adipose lipolysis has been shown to mitigate fat and muscle wasting [34]. Similarly, the activation of lipolytic pathways preceded muscle atrophy in our study, suggesting a causative role. These findings highlight the potential of targeting adipose lipolysis as a therapeutic strategy to prevent muscle wasting and cachexia progression and warrant further investigation. Additionally, adipocyte‐derived mediators, including pro‐inflammatory cytokines induced by smoking exposure, may also contribute to muscle atrophy. Previous studies have reported that, in addition to inflammatory cytokines, free fatty acids [S13] and adiponectin [S21] can induce myotube atrophy. Nevertheless, in this study, the identity of the principal adipose‐derived factors responsible for promoting myotube atrophy, as well as their underlying mechanisms of action, remains undefined, underscoring the need for further research. Moreover, our results indicate that adipose atrophy may contribute to muscle atrophy, and both temporal dynamics and in vitro evidence support the presence of crosstalk. However, direct in vivo validation is lacking, and the causal relationship remains to be elucidated. Future investigations should focus on the identification of key mediators and their functional modulation, which are expected to provide critical insights into the progression and potential reversal of muscle atrophy, thereby reinforcing the hypothesis advanced in this work. Furthermore, although we employed 3% CSE in our in vitro experiments, this concentration has generally been considered equivalent to approximately 10 cigarettes per day in humans [S22]. Nevertheless, direct extrapolation is difficult, and substantial differences may exist between the levels of smoking constituents encountered by human skeletal muscle in vivo and those encountered by myotubes in vitro. Furthermore, previous studies have demonstrated that muscle fibres exhibit functional changes during contraction [S23]. Thus, the functional effects of contraction, with the intricate interplay of multiple cell types in vivo, may lead to significant discrepancies between cellular experiments and physiological conditions, highlighting the need for further investigation.

Currently, the only established treatments for COPD‐related cachexia are nutritional interventions and rehabilitation programs [35, 36]. While these interventions improve nutrition, function and quality of life [35, 36], their effects on tissue composition are inconsistent, with some studies reporting only fat gain [37] and others reporting muscle gain as well [38] [S24]. Progressive loss of PM mass over time has been associated with increased mortality of current and former smokers, and this is independent of baseline BMI or disease severity [39]. These findings suggest that early interventions aimed at preserving muscle mass may have prognostic benefits. Our findings implicate early adipose tissue loss and browning as initiators of cachexia in COPD. This indicates that isolated fat loss before detectable muscle atrophy may represent a critical window for therapeutic intervention. Future studies should investigate whether such early cases can benefit from targeted treatments to prevent subsequent muscle wasting.

Some limitations should be acknowledged. First, our clinical cohort included few current smokers or female patients, while a substantial proportion presented with advanced emphysema and sarcopenia. Given that prior studies have reported sex‐related differences in COPD [S25] as well as distinctions based on smoking status [S26], caution is warranted when generalising the present findings to current smokers or female patients. Furthermore, as our in vivo experiments were conducted under continuous smoking exposure, rigorous comparison between the human and murine findings may not be feasible. Second, muscle strength was not evaluated in either the murine or human cohorts. Previous studies have demonstrated a correlation between muscle cross‐sectional area, as assessed using CT, and handgrip strength [40]. Additionally, smoking‐induced muscle weakness has been reported in humans [S27] and mice [23], suggesting the possibility that muscle weakness may also be present in this study. However, it remains unclear whether such weakness manifests early on or only at later stages, depending on smoking exposure and disease severity. Therefore, this point has been highlighted as an area warranting future investigation.

In conclusion, in patients with COPD, emphysema severity is independently associated with atrophy of the PM and EAT. Our findings show that adipose tissue atrophy precedes muscle wasting following cigarette smoke exposure, with smoke‐induced alterations in adipocyte phenotype potentially driving muscle atrophy. These findings support a central role for adipose tissue remodelling in the pathogenesis of cachexia in COPD and highlight adipose tissue as a promising therapeutic target for its prevention and management.

## Funding

The authors received no specific funding for this work.

## Ethics Statement

This study was registered with the University Hospital Medical Information Network (UMIN 000003470). The ethics committees of Keio University and its affiliated hospitals approved the study protocol. The study adhered to the principles outlined in the Declaration of Helsinki, which was adopted by the 59th WMA General Assembly, Seoul, Republic of Korea, in October 2008. All participants provided written informed consent for the use and publication of their data.

## Conflicts of Interest

The authors declare no conflicts of interest.

## Supporting information


**Figure S1:** Patient selection process in this study. Data were analysed from only patients who had undergone plain chest CT among those diagnosed with COPD by spirometry (defined as FEV₁/FVC < 0.7). COPD, chronic obstructive pulmonary disease; FEV_1_, forced expiratory volume in 1 s; FVC, forced vital capacity.
**Figure S2:** Pulmonary, adipose and skeletal muscle alterations in the elastase‐induced emphysema mouse model. (A) Representative H&E‐stained lung sections and evaluation of emphysematous changes (*n* = 5–6 per group). Scale bar: 100 μm. (B) Body weight changes over 3 weeks. (C) Representative macroscopic appearance of adipose tissue. Scale bar: 1 cm. (D) Comparison of adipose tissue weight (*n* = 19–20 per group). (E) Representative macroscopic appearance of skeletal muscle. Scale bar: 5 mm. (F) Comparison of skeletal muscle weight (n = 19–20 per group). ***p < 0.001; BAT, brown adipose tissue; WAT, white adipose tissue.

**Table S1:** Primer sequences.
**Table S2:** Correlation between cross‐sectional area of muscle and adipose tissue and airway indices in COPD patients.

**Data S1:** Supplementary Methods.

**Data S2:** Supplementary References.

## Data Availability

Data supporting the findings of the present study are available from the corresponding author upon reasonable request.
