# Supplementary material for: Adipose–Muscle Crosstalk in COPD Cachexia: Early Adipose Atrophy Drives Subsequent Muscle Wasting
Source: J Cachexia Sarcopenia Muscle. 2025 Dec 9;16(6):e70154. doi: 10.1002/jcsm.70154 (PMC12688405; doi:10.1002/jcsm.70154)
Supplement: Supplementary file 1 — Figure S1: Patient selection process in this study. Data were analysed from only patients who had undergone plain chest CT among those diagnosed with COPD by spirometry (defined as FEV₁/FVC < 0.7). COPD, chronic obstructive pulmonary disease; FEV1, forced expiratory volume in 1 s; FVC, forced vital capacity. Figure S2: Pulmonary, adipose and skeletal muscle alterations in the elastase‐induced emphysema mouse model. (A) Representative H&E‐stained lung sections and evaluation of emphysematous changes (n = 5–6 per group). Scale bar: 100 μm. (B) Body weight changes over 3 weeks. (C) Representative macroscopic appearance of adipose tissue. Scale bar: 1 cm. (D) Comparison of adipose tissue weight (n = 19–20 per group). (E) Representative macroscopic appearance of skeletal muscle. Scale bar: 5 mm. (F) Comparison of skeletal muscle weight (n = 19–20 per group). ***p < 0.001; BAT, brown adipose tissue; WAT, white adipose tissue. [file JCSM-16-e70154-s001.docx]

**Adipose–Muscle Crosstalk in COPD Cachexia: Early Adipose Atrophy Drives Subsequent Muscle Wasting**

Takashi Shimada^1^, Shotaro Chubachi^1*^, Keisuke Nishikawa^1^, Tetsuya Arai^1^, Hideto Iizuka^1^, Shiro Otake^1^, Kaori Sakurai^1^, Junko Hamamoto^1^, Mamoru Sasaki^2^, Tomoki Maetani^3^, Naoya Tanabe^3^, Katsunori Masaki^1^, Hiroki Kabata^1^, Jun Miyata^1^, Yoshitake Yamada^4^, Masahiro Jinzaki^4^, Hidetoshi Nakamura^5^, Koichiro Asano^6^, Koichi Fukunaga^1^

^1^ Division of Pulmonary Medicine, Department of Medicine, Keio University School of Medicine, Tokyo, Japan.

^2^ Department of Respiratory Medicine, Japan Community Health Care Organization (JCHO) Saitama Medical Center, Saitama, Japan.

^3^ Department of Respiratory Medicine, Graduate School of Medicine, Kyoto University, Kyoto, Japan.

^4^ Department of Radiology, Keio University School of Medicine, Tokyo, Japan.

^5^ Department of Respiratory Medicine, Saitama Medical University, Saitama, Japan.

^6^ Division of Pulmonary Medicine, Department of Medicine, Tokai University, School of Medicine, Kanagawa, Japan.

*** Corresponding Author:**

Shotaro Chubachi, MD, PhD

Division of Pulmonary Medicine, Department of Medicine, Keio University School of Medicine, 35 Shinanomachi, Shinjuku-ku, Tokyo 160-8582, Japan

Tel: +81-3-3353-1211

Fax: +81-3-3353-2502

Email: bachibachi472000@keio.jp


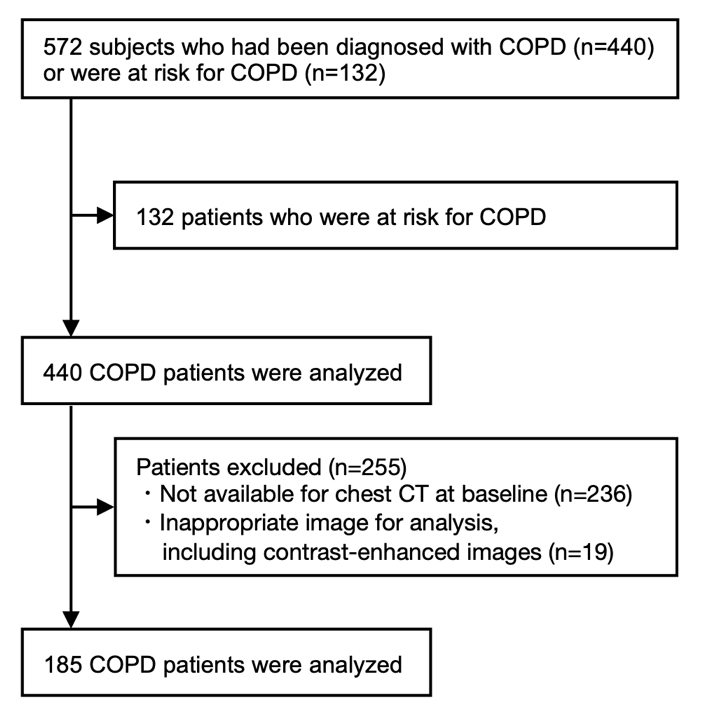


**Figure S1.** Patient selection process in this study. Data were analyzed from only patients who had undergone plain chest CT among those diagnosed with COPD by spirometry (defined as FEV₁/FVC < 0.7). COPD, chronic obstructive pulmonary disease; FEV_1_, forced expiratory volume in 1 s; FVC, forced vital capacity.


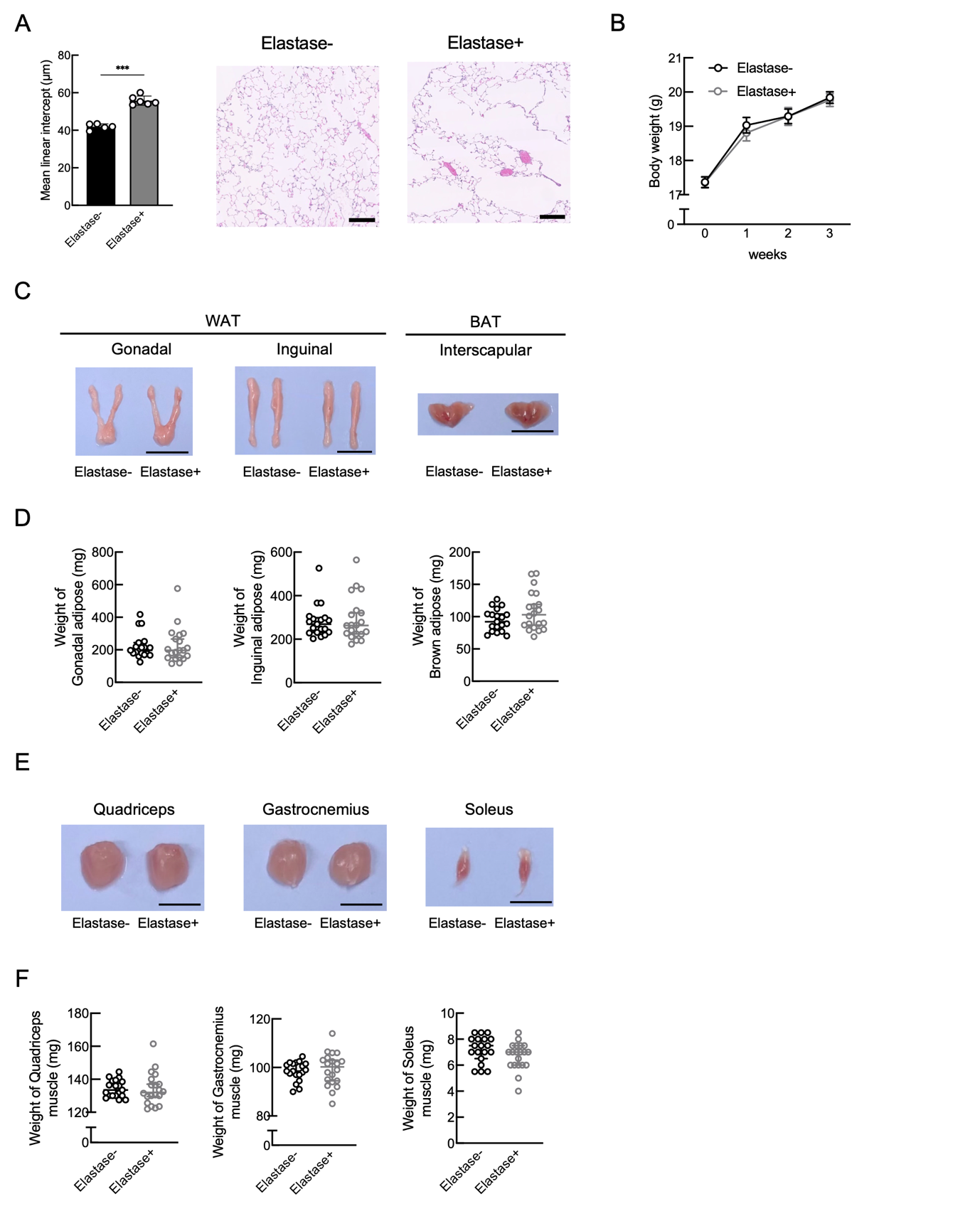


**Figure S2.** Pulmonary, adipose, and skeletal muscle alterations in the elastase-induced emphysema mouse model.

(a) Representative H&E-stained lung sections and evaluation of emphysematous changes (n = 5–6 per group). Scale bar: 100 µm. (b) Body weight changes over 3 weeks. (c) Representative macroscopic appearance of adipose tissue. Scale bar: 1 cm. (d) Comparison of adipose tissue weight (n = 19–20 per group). (e) Representative macroscopic appearance of skeletal muscle. Scale bar: 5 mm. (f) Comparison of skeletal muscle weight (n = 19–20 per group). ***, p<0.001; BAT, brown adipose tissue; WAT, white adipose tissue.
