# Supplementary material for: Adipose–Muscle Crosstalk in COPD Cachexia: Early Adipose Atrophy Drives Subsequent Muscle Wasting
Source: J Cachexia Sarcopenia Muscle. 2025 Dec 9;16(6):e70154. doi: 10.1002/jcsm.70154 (PMC12688405; doi:10.1002/jcsm.70154)
Supplement: Supplementary file 3 — Data S1: Supplementary Methods. [file JCSM-16-e70154-s004.docx]

**Adipose–Muscle Crosstalk in COPD Cachexia: Early Adipose Atrophy Drives Subsequent Muscle Wasting**

Takashi Shimada^1^, Shotaro Chubachi^1*^, Keisuke Nishikawa^1^, Tetsuya Arai^1^, Hideto Iizuka^1^, Shiro Otake^1^, Kaori Sakurai^1^, Junko Hamamoto^1^, Mamoru Sasaki^2^, Tomoki Maetani^3^, Naoya Tanabe^3^, Katsunori Masaki^1^, Hiroki Kabata^1^, Jun Miyata^1^, Yoshitake Yamada^4^, Masahiro Jinzaki^4^, Hidetoshi Nakamura^5^, Koichiro Asano^6^, Koichi Fukunaga^1^

^1^ Division of Pulmonary Medicine, Department of Medicine, Keio University School of Medicine, Tokyo, Japan.

^2^ Department of Respiratory Medicine, Japan Community Health Care Organization (JCHO) Saitama Medical Center, Saitama, Japan.

^3^ Department of Respiratory Medicine, Graduate School of Medicine, Kyoto University, Kyoto, Japan.

^4^ Department of Radiology, Keio University School of Medicine, Tokyo, Japan.

^5^ Department of Respiratory Medicine, Saitama Medical University, Saitama, Japan.

^6^ Division of Pulmonary Medicine, Department of Medicine, Tokai University, School of Medicine, Kanagawa, Japan.

*** Corresponding Author:**

Shotaro Chubachi, MD, PhD

Division of Pulmonary Medicine, Department of Medicine, Keio University School of Medicine, 35 Shinanomachi, Shinjuku-ku, Tokyo 160-8582, Japan

Tel: +81-3-3353-1211

Fax: +81-3-3353-2502

Email: bachibachi472000@keio.jp

**Supplemental Methods**

***Imaging Analysis of Human COPD***

A prospective observational cohort study was conducted at Keio University and its affiliated hospitals to investigate the management of comorbidities associated with chronic obstructive pulmonary disease (COPD). As previously reported, 572 individuals aged 40–91 years, including 440 diagnosed with COPD and 132 at risk, were enrolled between April 2010 and December 2012 by respiratory physicians [1]. For the present study, 185 patients with confirmed COPD based on spirometry and available plain chest CT data were included (Supporting Information Figure S1). All participants were clinically stable and had experienced no exacerbations for at least one month before enrollment. The study protocol was approved by the Ethics Committees of Keio University and its affiliated institutions. Written informed consent was obtained from all participants for data analysis and publication.

***Mice***

Female C57BL/6J mice (aged 7–8 weeks) were obtained from Sankyo Labo Service (Tokyo, Japan). The animals were housed in plastic cages under a 12-hour light/dark cycle.

***Cigarette Smoke-Induced Emphysema Mouse Model***

Mainstream cigarette smoke (CS) was generated from commercially available filtered cigarettes (Marlboro, tar 12 mg, nicotine 1.0 mg) and administered to mice via nasal inhalation as previously described [2]. The SIS-CS system (Shibata Scientific Technology, Tokyo, Japan), which includes a smoke generator (SG-300) and an inhalation chamber, was employed, accommodating up to 20 mice in body holders. The exposure settings were as follows: stroke volume, 15 mL; 10 puffs/min. The CS was diluted with compressed air to yield a total particulate matter concentration of 1,202 ± 196 mg/m³. Mice were exposed to CS for 60 minutes/day for 5 days/week for either 4 or 12 weeks. Age-matched control mice were exposed to ambient air under identical conditions.

***Elastase-Induced Emphysema Mouse Model***

Mice received a single intratracheal instillation of 1.5 units of porcine pancreatic elastase (Elastin Products, Owensville, MO) dissolved in 50 µL of sterile phosphate-buffered saline via a 22-gauge intravenous catheter [2]. Mice were euthanized 3 weeks after elastase administration for analysis.

***Histological Assessment of Mouse Organs***

Lungs were fixed by intratracheal infusion of 4% paraformaldehyde (PFA) at a constant pressure of 25 cmH₂O [3]. After removal, lungs were embedded in paraffin and stained with hematoxylin and eosin (H&E). Alveolar size was determined by quantifying the mean linear intercept (Lm). Soleus, gastrocnemius, quadriceps, perigonadal visceral fat, inguinal subcutaneous fat, and intrascapular brown adipose tissue were excised and fixed in 4% PFA. Following routine processing and paraffin embedding, sections were stained with H&E using standard protocols. Gastrocnemius muscle sections were stained with anti-laminin antibody (L9393; Sigma-Aldrich, St. Louis, MO, USA). Visceral fat sections were stained with anti-UCP1 antibody (ab10983; Abcam, Cambridge, UK) and anti-CD68 antibody (97778; Cell Signaling Technology, Danvers, MA, USA). The adipocyte area and muscle fiber cross-sectional area were quantified using ImageJ software.

***RNA Extraction and Reverse Transcription***

Total RNA was extracted using the RNeasy Lipid Tissue Mini Kit (74804; Qiagen, Hilden, NRW, Germany) for visceral adipose, the RNeasy Fibrous Tissue Mini Kit (74704; Qiagen) for gastrocnemius muscle, and the RNeasy Mini Kit (74104; Qiagen) for cultured cells. Reverse transcription was performed using ReverTra Ace® qPCR RT Master Mix (FSQ-201; TOYOBO, Tokyo, Japan).

***Protein Extraction and Western Blotting***

Protein concentrations were determined using the bicinchoninic acid (BCA) assay. Equal amounts of protein (10 µg per lane) were separated by SDS-PAGE and transferred onto polyvinylidene difluoride (PVDF) membranes. Membranes were incubated overnight at 4°C with primary antibodies, followed by incubation with secondary antibodies for 1 hour. The following antibodies were used: MuRF1 (ab172479; Abcam), Atrogin-1 (ab168372; Abcam), and GAPDH (2118; Cell Signaling Technology). Protein bands were visualized using Clarity Western ECL Substrate (Bio–Rad Laboratories, Hercules, CA, USA) and imaged with the LAS 4000 Mini System (GE Healthcare Life Sciences, Chicago, IL, USA). Densitometric analysis was performed using ImageJ software with normalization to GAPDH.

***Cell Culture***

Mouse preadipocytes (3T3-L1; CL-173; ATCC, Manassas, VA, USA) and skeletal muscle myoblasts (C2C12; CRL-1772; ATCC) were cultured in Dulbecco’s Modified Eagle Medium (DMEM) supplemented with 10% (v/v) fetal bovine serum (FBS), 100 U/mL penicillin, and 100 µg/mL streptomycin at 37°C in a humidified incubator with 5% CO₂. The 3T3-L1 cells were maintained in the medium until confluence. Two days after confluence, differentiation was induced using DMEM supplemented with 0.5 mM IBMX, 0.25 µM dexamethasone, and 10 µg/mL insulin for 3 days, subsequently followed by incubation in DMEM supplemented with 10 µg/mL insulin [4]. C2C12 myoblasts were cultured to near-confluence (~90%) and differentiated into myotubes by switching to DMEM supplemented with 2% horse serum with medium replacement every 48 hours.

***Preparation of Cigarette Smoke Extract (CSE)***

Smoke from a single cigarette was drawn through a constant airflow into a tube containing 5 mL of DMEM. The resulting CSE solution was sterilized by filtration through a 0.22 µm membrane filter and designated as 100% CSE.

**References**

1. Chubachi S, Nakamura H, Sasaki M, Haraguchi M, Miyazaki M, Takahashi S et al. Polymorphism of LRP5 gene and emphysema severity are associated with osteoporosis in Japanese patients with or at risk for COPD. Respirology 2015;20:286–95.

2. Sasaki M, Chubachi S, Kameyama N, Sato M, Haraguchi M, Miyazaki M et al. Evaluation of cigarette smoke-induced emphysema in mice using quantitative micro-computed tomography. Am. J. Physiol. Lung Cell. Mol. Physiol. 2015;308:L1039–45.

3. Takahashi S, Ishii M, Namkoong H, Hegab AE, Asami T, Yagi K et al. Pneumococcal infection aggravates elastase-induced emphysema via matrix metalloproteinase 12 overexpression. J. Infect. Dis. 2016;213:1018–30.

4. Reed BC, Lane MD. Insulin receptor synthesis and turnover in differentiating 3T3-L1 preadipocytes. Proc. Natl Acad. Sci. U. S. A. 1980;77:285–9.
