# Supplementary material for: Adipose–Muscle Crosstalk in COPD Cachexia: Early Adipose Atrophy Drives Subsequent Muscle Wasting
Source: J Cachexia Sarcopenia Muscle. 2025 Dec 9;16(6):e70154. doi: 10.1002/jcsm.70154 (PMC12688405; doi:10.1002/jcsm.70154)
Supplement: Supplementary file 4 — Data S2: Supplementary References. [file JCSM-16-e70154-s003.docx]

**Adipose–Muscle Crosstalk in COPD Cachexia: Early Adipose Atrophy Drives Subsequent Muscle Wasting**

Takashi Shimada^1^, Shotaro Chubachi^1*^, Keisuke Nishikawa^1^, Tetsuya Arai^1^, Hideto Iizuka^1^, Shiro Otake^1^, Kaori Sakurai^1^, Junko Hamamoto^1^, Mamoru Sasaki^2^, Tomoki Maetani^3^, Naoya Tanabe^3^, Katsunori Masaki^1^, Hiroki Kabata^1^, Jun Miyata^1^, Yoshitake Yamada^4^, Masahiro Jinzaki^4^, Hidetoshi Nakamura^5^, Koichiro Asano^6^, Koichi Fukunaga^1^

^1^ Division of Pulmonary Medicine, Department of Medicine, Keio University School of Medicine, Tokyo, Japan.

^2^ Department of Respiratory Medicine, Japan Community Health Care Organization (JCHO) Saitama Medical Center, Saitama, Japan.

^3^ Department of Respiratory Medicine, Graduate School of Medicine, Kyoto University, Kyoto, Japan.

^4^ Department of Radiology, Keio University School of Medicine, Tokyo, Japan.

^5^ Department of Respiratory Medicine, Saitama Medical University, Saitama, Japan.

^6^ Division of Pulmonary Medicine, Department of Medicine, Tokai University, School of Medicine, Kanagawa, Japan.

*** Corresponding Author:**

Shotaro Chubachi, MD, PhD

Division of Pulmonary Medicine, Department of Medicine, Keio University School of Medicine, 35 Shinanomachi, Shinjuku-ku, Tokyo 160-8582, Japan

Tel: +81-3-3353-1211

Fax: +81-3-3353-2502

Email: bachibachi472000@keio.jp

**Supplemental References**

1. McDonald MN, Wouters EFM, Rutten E, Casaburi R, Rennard SI, Lomas DA et al. It's more than low BMI: prevalence of cachexia and associated mortality in COPD. Respir. Res. 2019;20:100.
2. Mostert R, Goris A, Weling-Scheepers C, Wouters EF, Schols AM. Tissue depletion and health related quality of life in patients with chronic obstructive pulmonary disease. Respir. Med. 2000;94:859–67.
3. Engelen MP, Schols AM, Does JD, Wouters EF. Skeletal muscle weakness is associated with wasting of extremity fat-free mass but not with airflow obstruction in patients with chronic obstructive pulmonary disease. Am. J. Clin. Nutr. 2000;71:733–8.
4. Kaisari S, Rom O, Aizenbud D, Reznick AZ. Involvement of NF-κB and muscle specific E3 ubiquitin ligase MuRF1 in cigarette smoke-induced catabolism in C2 myotubes. Adv. Exp. Med. Biol. 2013;788:7–17.
5. Petersen AMW, Magkos F, Atherton P, Selby A, Smith K, Rennie MJ et al. Smoking impairs muscle protein synthesis and increases the expression of myostatin and MAFbx in muscle. Am. J. Physiol. Endocrinol. Metab. 2007;293:E843–8.
6. Agustí A, Morlá M, Sauleda J, Saus C, Busquets X. NF-kappaB activation and iNOS upregulation in skeletal muscle of patients with COPD and low body weight. Thorax 2004;59:483–7.
7. Barreiro E, Schols AM, Polkey MI, Galdiz JB, Gosker HR, Swallow EB et al. Cytokine profile in quadriceps muscles of patients with severe COPD. Thorax 2008;63:100–7.
8. De Brandt J, Beijers RJHCG, Chiles J, Maddocks M, McDonald MN, Schols AMWJ et al. Update on the etiology, assessment, and management of COPD cachexia: considerations for the clinician. Int. J. Chron. Obstruct. Pulmon. Dis. 2022;17:2957–76.
9. Henrot P, Blervaque L, Dupin I, Zysman M, Esteves P, Gouzi F et al. Cellular interplay in skeletal muscle regeneration and wasting: insights from animal models. J. Cachexia Sarcopenia Muscle 2023;14:745–57.
10. Rodin J. Weight change following smoking cessation: the role of food intake and exercise. Addict Behav. 1987;12:303–17.
11. Zimmermann R, Strauss JG, Haemmerle G, Schoiswohl G, Birner-Gruenberger R, Riederer M et al. Fat mobilization in adipose tissue is promoted by adipose triglyceride lipase. Science 2004;306:1383–6.
12. Han J, Meng Q, Shen L, Wu G. Interleukin-6 induces fat loss in cancer cachexia by promoting white adipose tissue lipolysis and browning. Lipids Health Dis. 2018;17:14.
13. Rupert JE, Narasimhan A, Jengelley DHA, Jiang Y, Liu J, Au E et al. Tumor-derived IL-6 and trans-signaling among tumor, fat, and muscle mediate pancreatic cancer cachexia. J. Exp. Med. 2021;218:e20190450.
14. Grant RW, Stephens JM.Stephens JM. Fat in flames: influence of cytokines and pattern recognition receptors on adipocyte lipolysis. Am. J. Physiol. Endocrinol. Metab. 2015;309:E205–13.
15. Jeanson Y, Carrière A, Casteilla L. A new role for browning as a redox and stress adaptive mechanism? Front. Endocrinol. (Lausanne) 2015;6:158.
16. Machado SA, Pasquarelli-do-Nascimento G, da Silva DS, Farias GR, de Oliveira Santos I, Baptista LB et al. Browning of the white adipose tissue regulation: new insights into nutritional and metabolic relevance in health and diseases. Nutr. Metab. (Lond.) 2022;19:61.
17. Kir S, White JP, Kleiner S, Kazak L, Cohen P, Baracos VE et al. Tumour-derived PTH-related protein triggers adipose tissue browning and cancer cachexia. Nature 2014;513:100–4.
18. Kir S, Komaba H, Garcia AP, Economopoulos KP, Liu W, Lanske B et al. PTH/PTHrP receptor mediates cachexia in models of kidney failure and cancer. Cell Metab. 2016;23:315–23.
19. Bodine SC, Baehr LM. Skeletal muscle atrophy and the E3 ubiquitin ligases MuRF1 and MAFbx/atrogin-1. Am. J. Physiol. Endocrinol. Metab. 2014;307:E469–84.
20. Degens H. The role of systemic inflammation in age-related muscle weakness and wasting. Scand. J. Med. Sci. Sports 2010;20:28–38.
21. Ito R, Higa M, Goto A, Aoshima M, Ikuta A, Ohashi K et al. Activation of adiponectin receptors has negative impact on muscle mass in C2C12 myotubes and fast-type mouse skeletal muscle. PLOS One 2018;13:e0205645.
22. Su Y, Han W, Giraldo C, De LY, Block ER. Effect of cigarette smoke extract on nitric oxide synthase in pulmonary artery endothelial cells. Am J Respir Cell Mol Biol. 1998;19:819–25.
23. Lambernd S, Taube A, Schober A, Platzbecker B, Görgens SW, Schlich R et al. Contractile activity of human skeletal muscle cells prevents insulin resistance by inhibiting pro-inflammatory signalling pathways. Diabetologia. 2012;55:1128–39.
24. Pison CM, Cano NJ, Chérion C, Caron F, Court-Fortune I, Antonini MT et al. Multimodal nutritional rehabilitation improves clinical outcomes of malnourished patients with chronic respiratory failure: a randomised controlled trial. Thorax 2011;66:953–60.
25. Aryal S, Diaz-Guzman E, Mannino DM. COPD and gender differences: an update. Transl Res. 2013;162:208–18.
26. Zhang J, Lin XF, Bai CX. Comparison of clinical features between non-smokers with COPD and smokers with COPD: a retrospective observational study. Int J Chron Obstruct Pulmon Dis. 2014;9:57–63.
27. Kok MO, Hoekstra T, Twisk JW. The longitudinal relation between smoking and muscle strength in healthy adults. Eur Addict Res. 2012;18:70–5.
